# Bayesian variable selection and survival modeling: assessing the Most important comorbidities that impact lung and colorectal cancer survival in Spain

**DOI:** 10.1186/s12874-022-01582-0

**Published:** 2022-04-03

**Authors:** Francisco Javier Rubio, Danilo Alvares, Daniel Redondo-Sanchez, Rafael Marcos-Gragera, María-José Sánchez, Miguel Angel Luque-Fernandez

**Affiliations:** 1 Department of Statistical Science, University College London, London, UK; 2 Department of Statistics, Pontificia Universidad Católica de Chile, Macul, Chile; 3 Non-communicable Disease and Cancer Epidemiology Group, Instituto de Investigación Biosanitaria ibs.GRANADA, Granada, 18012 Spain; 4 Centro de Investigación Biomédica en Red de Epidemiología y Salud Pública (CIBERESP), Madrid, 28029 Spain; 5 Escuela Andaluza de Salud Pública (EASP), Granada, 18011 Spain; 6 Epidemiology Unit and Girona Cancer Registry, Oncology Coordination Plan, Department of Health, Autonomous Government of Catalonia, Catalan Institute of Oncology, Girona, Spain; 7 Descriptive Epidemiology, Genetics and Cancer Prevention Group, Biomedical Research Institute (IDIBGI), Girona, Spain; 8 Department of Preventive Medicine and Public Health, University of Granada, Granada, 18071 Spain; 9 Inequalities in Cancer Outcomes Network, Department of Non-communicable Disease Epidemiology, London School of Hygiene and Tropical Medicine, London, UK

**Keywords:** Bayesian variable selection, Cancer survival, Comorbidities, Conditional effects, Marginal effects

## Abstract

Cancer survival represents one of the main indicators of interest in cancer epidemiology. However, the survival of cancer patients can be affected by several factors, such as comorbidities, that may interact with the cancer biology. Moreover, it is interesting to understand how different cancer sites and tumour stages are affected by different comorbidities. Identifying the comorbidities that affect cancer survival is thus of interest as it can be used to identify factors driving the survival of cancer patients. This information can also be used to identify vulnerable groups of patients with comorbidities that may lead to worst prognosis of cancer. We address these questions and propose a principled selection and evaluation of the effect of comorbidities on the overall survival of cancer patients. In the first step, we apply a Bayesian variable selection method that can be used to identify the comorbidities that predict overall survival. In the second step, we build a general Bayesian survival model that accounts for time-varying effects. In the third step, we derive several posterior predictive measures to quantify the effect of individual comorbidities on the population overall survival. We present applications to data on lung and colorectal cancers from two Spanish population-based cancer registries. The proposed methodology is implemented with a combination of the R-packages mombf and rstan. We provide the code for reproducibility at https://github.com/migariane/BayesVarImpComorbiCancer.

## Introduction

Selecting the set of patient and tumour characteristics that better predict the survival probability of cancer patients is of primary interest in cancer epidemiology as this information can be used to inform policymakers, clinicians, and epidemiologists (see Michalopoulou et al. [1] for a discussion) [2]. Moreover, quantifying the variable importance (in our context, for predicting survival) can be used to identify the most relevant patient’s characteristics that may affect their prognosis. At the population level, the information about risk factors in cancer patients can be used to stratify groups of patients at higher risk. Information about comorbidities from cancer registries is typically limited, but recent computational algorithms have allowed the identification of comorbidities at the population level using patients’ hospital records [3–5].

Several methods can be applied to perform variable selection (e.g., selection of comorbidities in prognostic cancer epidemiology) in the frequentist and Bayesian frameworks. Briefly, in a frequentist framework, it is common to use stepwise selection methods based on information criteria such as Akaike information criterion (AIC), Bayesian information criterion (BIC), or deviance information criterion (DIC). Two limitations of this approach are their poor performance in terms of correct model selection for finite samples and potential multiplicity problems [6]. Alternatively, penalised likelihood methods have become popular in survival analysis. These include using LASSO, Ridge, and Elastic nets penalties for Proportional Hazards (PH) and Accelerated Failure Time (AFT) models [7]. However, penalised methods do not allow for quantifying the uncertainty about the selected models, and their performance can be affected by high correlations between the variables. Several Bayesian variable selection methods have been proposed based on different combinations of priors and survival models (we refer the reader to Rossel and Rubio [8] for a thorough overview of these methods). These methods allow for quantifying the uncertainty about the selection models, as posterior probabilities can be assigned to each model, and to quantify the variable importance through the calculation of posterior inclusion probabilities (PIP), which can be interpreted as a measure of the importance of individual variables in explaining the response [9].

In practice, we are interested in conducting statistical inference and drawing conclusions from the selected variables. Thus, the post-selection inference is a step as necessary as variable selection. The natural post-selection steps are modelling the survival response based on the selected variables and quantifying the effect of the selected variables on the survival probability. There is an extensive catalog of survival regression models, which are typically formulated in terms of the hazard function and aim to include effects that play a role on the time and hazard scales. We refer the reader to Rubio et al. [10] for a review on hazard regression models and a detailed discussion on the parametric models used in this paper (which we will refer to as General Hazards models). General Hazards (GH) models allow for incorporating hazard-level and time-level effects (i.e., effects that play a role on the hazard and time scales) while avoiding the need for numerical integration. This class of models includes, as particular cases, the PH, the AFT, and Accelerated Hazards (AH) models. This tractability and interpretability are helpful in survival data modelling as it allows the user to specify the roles of the covariates in the model. Once the survival model is fitted, it is useful to produce model summaries that help the user understand the effect of the variables on the survival probability. Several conditional and marginal measures can be used to assess these effects. Briefly, conditional effect measures aim at quantifying the effect of a variable in the observed population by comparing the survival curves, at specific time points, associated to individuals with and without a characteristic of interest (e.g. a comorbidity). Conditional measures in the context of survival analysis include the conditional risk differences and hazard ratios [11], the attributable risk, and the attributable survival of a particular covariate pattern [12]. In contrast, marginal measures aim at quantifying the effect of a characteristic of interest in the entire population. These include marginal effects based on the survival function, the marginal risk differences, and the restricted mean survival time [12]. These concepts will be described in the following sections.

Based on the use of Bayesian methodology, we aim to provide the end-user with guidelines to address the research questions about: (i) how to select the most important variables (i.e., comorbidities) that affect the survival of cancer patients, accounting for the modeling uncertainty; and (ii) how to quantify the effect of the selected variables using conditional and marginal measures of association. We also aim to illustrate the proposed methodology using real data on comorbidities and survival times of cancer patients.

The remainder of the paper is organised as follows. Motivating examples section presents a discussion of the data sets that motivated our work and that will be used in our applications. Methodological framework section introduces the three steps in the proposed Bayesian setting, including variable selection, survival modelling, and the calculation of summary measures. Results section presents two applications using population-based data on colorectal and lung cancer in Spain. We discuss the use and interpretation of the proposed methodology and explore the conclusions obtained by stratifying the data by grouping tumour stages (using their biological and clinical differences). Since the variable containing information about the tumour stage contains missing observations, we also present a sensitivity analysis of the results obtained by using complete cases *vs.* imputing the missing covariates.

## Motivating examples

The methodological framework proposed in this paper is motivated by timely and recent epidemiological questions. This section describes the data sets that motivate these research questions.

Worldwide, lung and colorectal cancer (CRC) are currently among the three most frequent anatomical locations regarding incidence and mortality [13]. Cancer survival indicators for lung and CRC varies widely between countries. For instance, 5-year age-standardised net survival for lung cancer patients diagnosed during 2010–2014 was high in Japan (33%), it ranged between 20–30% in Canada, the USA, and other European countries, but survival was below 10% in Thailand, Brazil, Bulgaria and India [14].

We analyse data from a population-based cohort study including patients diagnosed with CRC and primary lung and bronchus cancer incident cases diagnosed from 1^*st*^ January 2011 to 31^*st*^ December 2012 in two Spanish population-based cancer registries - Girona and Granada. The diagnoses were based on codes C18-C21 for CRC, including anal cancers, and C34.0, C34.1, C34.2, C34.3, C34.8, C34.9, for lung cancer according to the International Classification of Diseases for Oncology, 3^*rd*^ Edition [15]. The entry date of each patient into the cohort was defined as the date of cancer diagnosis, and their exit date was defined as the date of death or the date at 6 years after their cancer diagnosis for CRC cancer and 8-years for lung cancer, whichever occurred first.

Data on cancer stage at diagnosis (TNM staging system, 7^*th*^ edition [16]), comorbidities, and sex, were obtained retrospectively from patients’ medical records. The data collection followed a detailed protocol from the European High-Resolution studies collaboration (TRANSCAN-HIGHCARE project within the ERA-Net [17]). The vital status was assessed using the national death registry of the Spanish Ministry of Health. Comorbidities were assessed using the codes from the International Classification of Diseases, 10^*th*^ Revision [18]. All retrospectively recorded comorbidities in the medical records were included except for those diagnosed within 6 months before cancer diagnosis to prevent including cancer-related comorbidities [3].

## Methodological framework

In this section, we describe the proposed Bayesian methodological framework. Briefly,we propose the following steps:

1. In step one, we select the relevant variables that predict the survival times using an AFT model coupled with two types of priors [8]. The idea behind this methodology consists of selecting variables in AFT models using priors specifically developed to improve the finite-sample performance and consistency of selection. These include, the *g*-Zellner prior (*π*_*L*_) as well as a non-local prior (*π*_*M*_) [8], which help enforce parsimony. Variable selection is conducted using a formal Bayesian approach based on posterior probabilities of the different models and assessing the importance of the selected variables via calculating their PIPs. We perform this step using the R-package mombf (version 3.0.4) [19].
2. The second step consists of the model building based on a rich family of hazard regression models that contains the most common survival models (PH, AFT, and AH) as particular cases [10]. We fit this Bayesian survival model using the R-package rstan (version 2.21.2) [20].
3. In step three, we provide several conditional and marginal posterior predictive measures that allow for quantifying the effects of the selected individual comorbidities on the population survival.

We emphasise that these steps can be conducted on the entire population or stratified subpopulations of interest. For instance, one could think of stratifying the population into early and late tumour stages as there are biological and clinical differences between advanced and early stages of cancer [21] or sex [22]. We explore and discuss this idea further in our applications. Note that the software can be easily adapted to other data sets, and the code for running the three steps in the proposed methodological approach is available at the GitHub repository: https://github.com/migariane/BayesVarImpComorbiCancer.

### Step 1: Bayesian variable selection

Throughout, let $o_{i} \in \mathbb {R}_{+}$ be the time to event of interest, and $\mathbf {x}_{i}= (x_{i1},\ldots,x_{ip})^{\top } \in {\mathbb R}^{p}$ be the corresponding vector of covariates containing all of the available patient characteristics, for individuals *i*=1,…,*n*. Let $c_{i} \in \mathbb {R}_{+}$ denote the right-censoring times, such that one only observes the times *t*_*i*_= min{*o*_*i*_,*c*_*i*_}. Denote by *δ*_*i*_=I(*o*_*i*_<*c*_*i*_) the vital status observation *i*, and define *y*_*i*_= min{log(*o*_*i*_), log(*c*_*i*_)} the observed log-times, **y**=(*y*_1_,…,*y*_*n*_),***δ***=(*δ*_1_,…,*δ*_*n*_), and the number of uncensored individuals $n_{o}= \sum _{i=1}^{n} \delta _{i}$.

The variable selection step is based on the proposed methodology in Rossell and Rubio [8], which we briefly detail below. The aim here is to select the important variables that explain survival. To do so, we introduce an inclusion indicator ***γ***=(*γ*_1_,…,*γ*_*p*_), where

$$\gamma_{j} = \begin{array}{ll} 0,& \text{if}\ \beta_{j}= 0, \\ 1,& \text{if}\ \beta_{j} \neq 0, \end{array} $$

and *j*=1,…,*p*. That is, ***γ***=(*γ*_1_,…,*γ*_*p*_) determines which covariates are included in the model. For the selection step, we adopt an AFT model. This model assumes a log-linear regression structure:

$$ \log(o_{i}) = \mathbf{x}_{i}^{\top}\boldsymbol{\beta}_{\gamma} + \epsilon_{i}, $$

where *ε*_*i*_ are independent across *i*=1,…,*n* with mean *E*(*ε*_*i*_)=0 and variance *Var*(*ε*_*i*_)=*σ*^2^. For simplicity, we will assume that *ε*_*i*_∼*i*.*i*.*d*.*N*(0,*σ*^2^). Let **X**,**X**_*o*_,**X**_*c*_ denote the design matrices associated to entire sample, the uncensored survival times, and the censored survival times, respectively. Throughout, we will assume that **X**_*o*_ has full column rank.

In order to obtain a log-concave likelihood function, which in turns improves the performance of optimisation methods, Rossell and Rubio [8] adopt the reparameterisation ***α***_***γ***_=***β***_***γ***_/*σ*, and *τ*=1/*σ*. The log-likelihood under this parameterisation can be written as follows

$$\begin{array}{@{}rcl@{}} \ell(\boldsymbol{\alpha}_{\gamma},\tau) &=& -\frac{n_{o}}{2} \log\left(\frac{2\pi}{\tau^{2}}\right) - \frac{1}{2} \sum_{\delta_{i}=1}\left(\tau y_{i}-\mathbf{x}_{i}^{\top}\boldsymbol{\alpha}_{\gamma}\right)^{2} \\&&+ \sum_{\delta_{i}=0} \log \left\{\Phi\left(\mathbf{x}_{i}^{\top}\boldsymbol{\alpha}_{\gamma} - \tau y_{i}\right)\right\}. \end{array} $$

We adopt the following priors for the model parameters [8]:

$$\begin{array}{@{}rcl@{}} {\pi_{L}(\boldsymbol{\alpha}_{\gamma},\tau)} &=& \prod_{\gamma_{j} = 1}N\left(\alpha_{j}; 0, g_{L} n/ \left(x_{j}^{\top} x_{j}\right)\right) \pi(\tau),\\ \pi_{M}(\boldsymbol{\alpha}_{\gamma},\tau) &=& \prod_{\gamma_{j}= 1} \frac{\alpha_{j}^{2}}{g_{M}} N\left(\alpha_{j}; 0, g_{M}\right) \pi(\tau), \end{array} $$

where *π*(*τ*)=2*τ*^−3^IG(*τ*^−2^;*a*_*τ*_/2,*b*_*τ*_/2), and IG denotes the inverse gamma density, and $g_{L},g_{M},a_{\gamma },b_{\tau } \in \mathbb {R}_{+}$ are given dispersion parameters. The hyperparameter elicitation step is open to several choices, but here we discuss two specific options. One option, that we adopt by default in our applications, is to adopt the hyperparameters that induce a unit information prior [23], which can be interpreted as prior containing as much information as a single observation. This prior can be specified in the R package mombf using the option taustd = 1, as specified in the R code provided. Another option corresponds to the recommendations in [8], which are based on penalising the inclusion of small effects. To this aim [8], propose the choice *g*_*M*_=0.192,*g*_*L*_=1, and *a*_*τ*_=*b*_*τ*_=1, which assign low probability to small effects with $\phantom {\dot {i}\!}e^{\beta _{j}}<1.15$. Thus, penalising effect sizes that may be deemed irrelevant in practice. Our results and conclusions were robust to both choices.

We also adopt a Beta-Binomial prior on the different models [8]:

$$ \pi(\boldsymbol{\gamma})=\text{BetaBin}(p_{\gamma};p,a_{1},b_{1}),  $$

where BetaBin(*z*;*p*,*a*,*b*) is the probability of *z* successes under a Beta-Binomial distribution with *p* trials and parameters (*a*,*b*).

Based on this formulation, we obtain the following model posterior probabilities

1 $$ \begin{aligned} \pi(\boldsymbol{\gamma} \mid y) = \frac{p(y \mid \boldsymbol{\gamma})\pi(\boldsymbol{\gamma})}{\sum_{\boldsymbol{\gamma}} p(y \mid \boldsymbol{\gamma})\pi(\boldsymbol{\gamma})} = \left(1 + \sum_{\boldsymbol{\gamma}' \neq \boldsymbol{\gamma}} B_{\boldsymbol{\gamma}', \boldsymbol{\gamma}} \frac{\pi(\boldsymbol{\gamma}')}{\pi(\boldsymbol{\gamma})} \right)^{-1}, \end{aligned}  $$

where *π*(***γ***) is the model prior probability, $\phantom {\dot {i}\!}B_{\boldsymbol {\gamma }',\boldsymbol {\gamma }}= p(y \mid \boldsymbol {\gamma }')/p(y \mid \boldsymbol {\gamma })$ the Bayes factor between $\phantom {\dot {i}\!}(\boldsymbol {\gamma }',\boldsymbol {\gamma })$ and

$$p(y\mid \boldsymbol{\gamma})= \int p(y \mid \boldsymbol{\alpha}_{\boldsymbol{\gamma}},\tau) \pi(\boldsymbol{\alpha}_{\boldsymbol{\gamma}},\tau \mid \boldsymbol{\gamma}) d\boldsymbol{\alpha}_{\boldsymbol{\gamma}} d\tau, $$

the integrated likelihood *p*(*y*∣***α***_***γ***_,*τ*) with respect to a prior density *π*(***α***_***γ***_,*τ*∣***γ***). This integrated likelihood is calculated with a Laplace approximation in Rossell and Rubio [8].

One option for model selection consists of choosing the model with highest posterior probability $\widehat {\boldsymbol {\gamma }} = \operatorname {argmax}_{\boldsymbol {\gamma }}\pi (\boldsymbol {\gamma } \mid y)$. However, the 2^*p*^ models are often assigned low probabilities for models with many covariates. Thus, the posterior model probabilities are combined with marginal PIPs of the variables [24].

$$\text{PIP}(\gamma_{j} = 1 \mid y) = \sum_{\gamma_{j} = 1}\pi(\boldsymbol{\gamma} \mid y), $$

which represents the sum of the posterior probabilities of the models that contain the variable of interest *γ*_*i*_=1. This quantity can be used to assess the individual variable importance and has an excellent interpretation as it has a formal connection with a probability (thus, naturally bounded on the interval [0,1]). It is often helpful to look at the model containing those variables with PIP larger than 0.5, which can build a survival model for the selected variables. This methodology is implemented in the R-package mombf (version 3.0.4) [19].

### Step 2: modelling using a Bayesian parametric Hazard regression

Once the set of important variables, say $\mathbf {z}_{i} \in {\mathbb R}^{q}$, are selected, we develop a richer survival model that allows for the inclusion of time-dependent effects, hazard-level effects, as well as a parametric baseline hazard based on flexible distributions that can capture a variety of shapes of interest in practice [10]. More specifically, we consider the general hazard structure

2 $$ h_{GH}(t\mid \mathbf{z}_{i}, \boldsymbol{\xi}, \tilde{\boldsymbol{\theta}}, \boldsymbol{\theta}) = h_{0}\left(t \exp\{\tilde{\mathbf{z}}_{i}^{\top}\tilde{\boldsymbol{\theta}} \} \mid \boldsymbol{\xi}\right)\exp\{\mathbf{z}_{i}^{\top} \boldsymbol{\theta} \},  $$

where *h*_0_(·∣***ξ***) is a parametric baseline hazard with vector parameter $\boldsymbol {\xi } \in \Xi, \tilde {\boldsymbol {\theta }} \in {\mathbb R}^{\tilde {q}}$ represents the regression coefficients associated to the time-dependent effects $\tilde {\mathbf {z}}_{i} \in {\mathbb R}^{\tilde {q}}, \boldsymbol {\theta } \in {\mathbb R}^{q}$ are the regression coefficients associated to the hazard-level effects **z**_*i*_. Typically, $\tilde {\mathbf {z}}_{i} \subset \mathbf {z}_{i}$. This hazard structure contains, as particular cases, the Proportional Hazards (PH, $\tilde {\boldsymbol {\theta }}=0$), Accelerated Hazards (AH, ***θ***=0), and Accelerated Failure Time (AFT, $\tilde {\boldsymbol {\theta }} = \boldsymbol {\theta }$ and $\tilde {\mathbf {z}}_{i} = \mathbf {z}_{i}$) models. The baseline hazard can be chosen to be the hazard associated with the 3-parameter Power Generalised Weibull or Generalised Gamma distributions (see: https://github.com/FJRubio67/Distributions) which can capture the basic shapes of interest in practice (increasing, decreasing, unimodal, and bathtub) [25]. Simpler 2-parameter distributions such as the Log-Normal, Log-Logistic, or Gamma distributions can be used as well. In our implementation, we allow for various combinations of baseline hazards. Moreover, since the implementation is done in rstan (version 2.21.2) [20], this allows for selecting the best survival model (i.e., the combination of hazard structure and parametric baseline hazard) using posterior model probabilities calculated with the R-package bridgesampling (version 1.1-2) [26]. Since the aim of this step consists of inference on the model parameters, we adopt weakly informative priors. This requires a case-by-case analysis, but we generally adopt half-Cauchy priors for scale and shape parameters of the baseline hazard and normal priors with large variance for the regression coefficients. We point out that other priors could be used as well (see Alvares and Rubio [27] for a discussion) and that our implementation in rstan allows for easily changing this choice. In our applications, we only include comorbidities in **z** as we do not expect binary covariates to have a time-varying effect (based on clinician discussions). We only consider hazard-level effects of the comorbidities as these are binary variables.

### Step 3: summary measures

The Bayesian model fitted in Step 2 will now be used to explore the effect of the comorbidities using several predictive posterior conditional and marginal measures of association between the vector of selected comorbidities and cancer survival. We start by calculating the conditional posterior hazard ratios (HR) of the comorbidities, which are simply obtained as the exponential of the corresponding estimates of the coefficients, based on the hazard structure (2). We now introduce two survival functions representing posterior predictive conditional and marginal population survival functions associated with a comorbidity of interest. Let $\left (\boldsymbol {\xi }^{(j)},\tilde {\boldsymbol {\theta }}^{(j)},\boldsymbol {\theta }^{(j)}\right), j=1,\dots,M$, be a posterior sample of the model parameters. The predictive posterior survival function conditional on *z*_*i*,*k*_=*r* is defined by:

3 $$ \begin{aligned} \text{CS}(t, k, r)\! = \frac{1}{n_{r} M} \sum_{j=1}^{M}\sum_{z_{i,k} = r} \exp\left\{ -H_{GH}\left(t\mid \mathbf{z}_{i}, \boldsymbol{\xi}^{(j)},\tilde{\boldsymbol{\theta}}^{(j)},\boldsymbol{\theta}^{(j)}\right)\right\},  \end{aligned}  $$

where *n*_*r*_ are the number of individuals with *z*_*i*,*k*_=*r*,*r*∈{0,1}, and *H*_*GH*_ represents the cumulative hazard function associated to (2), which does not require numerical integration.

Now, let *z*_*i*,*k*_ be comorbidity of interest for patient *i*, and let **z**_*i*,−*k*_ and $\tilde {\mathbf {z}}_{i,-k}$ be the vectors of covariates after removing the covariate of interest *z*_*i*,*k*_. The predictive posterior marginal survival function associated to assuming that comorbidities *z*_*i*,*k*_=*r*,*r*=0,1 and *i*=1,…,*n*, is defined as follows:

4 $$\begin{array}{*{20}l} \text{MS}(t, k, r) &= \frac{1}{n M} \sum_{j=1}^{M}\sum_{i=1}^{n} \exp\left\{ -H_{GH}\left(t\mid \mathbf{z}_{i,-k}, z_{i,k} \right.\right.\\&\left.\left.= r, \boldsymbol{\xi}^{(j)},\tilde{\boldsymbol{\theta}}^{(j)},\boldsymbol{\theta}^{(j)}\right)\right\}. \end{array} $$

These two predictive survival functions (i.e., conditional and marginal survival functions) represent the main ingredient in the following measures of the effect of the *r*th comorbidity on the population survival function. Note that we do not favour using a specific measure, but we consider that both can provide complementary information.

From (3) and (4) we can compute the following conditional or marginal measures:

(i) The posterior predictive conditional effect (PPCE) is an effect on the conditional survival function. This is simply obtained as the difference of the conditional survival functions at time *t* for the comorbidity of interest:
  $$\text{PPCE}(t, k) = \text{CS}(t, k, 0) - \text{CS}(t, k, 1). $$
  This function is interpreted at a population level. It represents the change in survival in the group of patients having particular comorbidity (*k*) compared to the group of cancer patients who do not have that comorbidity. A positive PPCE quantifies the effect of comorbidity in reducing survival in the exposed subpopulation compared to the non-exposed sub-population.
(ii) The posterior predictive attributable survival (PPAS) function:
  $$\begin{aligned} \text{PPAS}(t, k) = \pi_{r}\frac{\text{CS}(t, k, 0) - \text{CS}(t, k, 1)}{\text{CS}(t, k, 0)} = \pi_{r}\left[ 1 - \frac{ \text{CS}(t, k, 1)}{\text{CS}(t, k, 0) }\right], \end{aligned} $$
  where *π*_*r*_ is the proportion of exposed individuals in the population to the *r*th comorbidity. This is a time-varying weighted relative difference of the conditional survival functions, which represents the ratio of the change in survival in the exposed *vs.* non-exposed groups (for specific comorbidity) and the conditional survival of the non-exposed group.
(iii) The posterior predictive attributable risk (PPAR) function:
  $$\begin{aligned} \text{PPAR}(t, k) = \frac{\pi_{r}\left[\text{CS}(t, k, 0) - \text{CS}(t, k, 1)\right]}{\pi_{r}\left[\text{CS}(t, k, 0) - \text{CS}(t, k, 1)\right] + 1 - \text{CS}(t, k, 0)}. \end{aligned} $$
  This is also a time-varying weighted version of the PPCE. This function can be interpreted as the portion of the detrimental outcome rate attributable to comorbidity in our context. We refer the reader to Cox, Chu and Muñoz [28] for a more extensive discussion on the effect measures (ii) and (iii).
(iv) The posterior predictive marginal effect (PPME) at time *t* is defined as
  $$\text{PPME}(t, k) = \text{MS}(t, k, 0) - \text{MS}(t, k, 1). $$
  This function represents the change in survival in the entire population induced from having a particular comorbidity (*k*). This measure takes values in (−1,1) and is interpreted as the marginal survival probability difference between having or not a particular comorbidity in the entire population [29].
(v) The posterior predictive restricted mean survival time (RMST)
  $$\text{RMST}(t^{*}, k,r) = \int_{0}^{t^{*}} \text{MS}(t, k,r)dt. $$
  The RMST represents the area under the marginal survival curve MS(*t*,*k*,*r*) between time 0 and a horizon time *t*^∗^, which does not rely on the proportionality of hazards assumption. The RMST can be interpreted as the average time free from an event (dead) between time 0 and the horizon time *t*^∗^. Comparing the functions RMST(*t*^∗^,*k*,*r*) for different time horizons, and *r*=1*vs*.*r*=0 helps quantifying the effect of a comorbidity on survival along a time period [30, 31].

## Results

In this section, we present the results from two applications of the proposed methodology in the context of assessing the interplay of comorbidities with colorectal and lung cancer survival. The data sets used in these applications were described in Motivating examples section, so we focus on presenting and discussing results. In addition to the proposed three steps in our methodology, we address the problem of missing data, which mainly affect the variable containing information about the tumour stage. We perform a sensitivity analysis by replicating the three steps after imputing the missing variables (stage and comorbidities for CRC and lung cancer, and smoking status for lung cancer). We assumed data were missing at random (i.e., we assume that stage is missing at random conditional on the information provided by the covariates gender, age, and comorbidities. Thus, we implicitly assume the probability of missingness is independent of the (possibly missing) yes/no value of stage after adjusting for (conditional on) sex, age, and comorbidities). We use the R-package mice (version 3.13.0) to implement a multivariate imputation via Chained Equations [32]. We imputed five data sets. The imputation model included the Nelson-Aalen estimator of the cumulative hazard evaluated at the exit time, age, sex, and the vital status indicator. Variable importance results after the multiple imputations of the missing stage were consistent and selected the same covariates.

### Results: CRC data analysis by stage

Among 1,061 CRC patients, 60.7% were female, and 20.7% of the patients had stage IV. The proportion of CRC women with stage IV was approximately two times higher than the proportion of men with stage IV (i.e., 62.2% vs. 37.8%). The median age at CRC diagnosis was 71 years with an interquartile range (IQR) of 17 years. The pattern of comorbidities was similar by CRC stage. The most common comorbidities among stages I-III CRC patients were diabetes (25.2%), chronic obstructive pulmonary disease (COPD) (18.5%), and heart failure (14.5%). Among stage IV patients, we observed diabetes (20.4%), heart failure (16.2%), and COPD (14.2%). There was 5.7% of the missing stage (Supplementary Table 1).

**Fig. 1:**
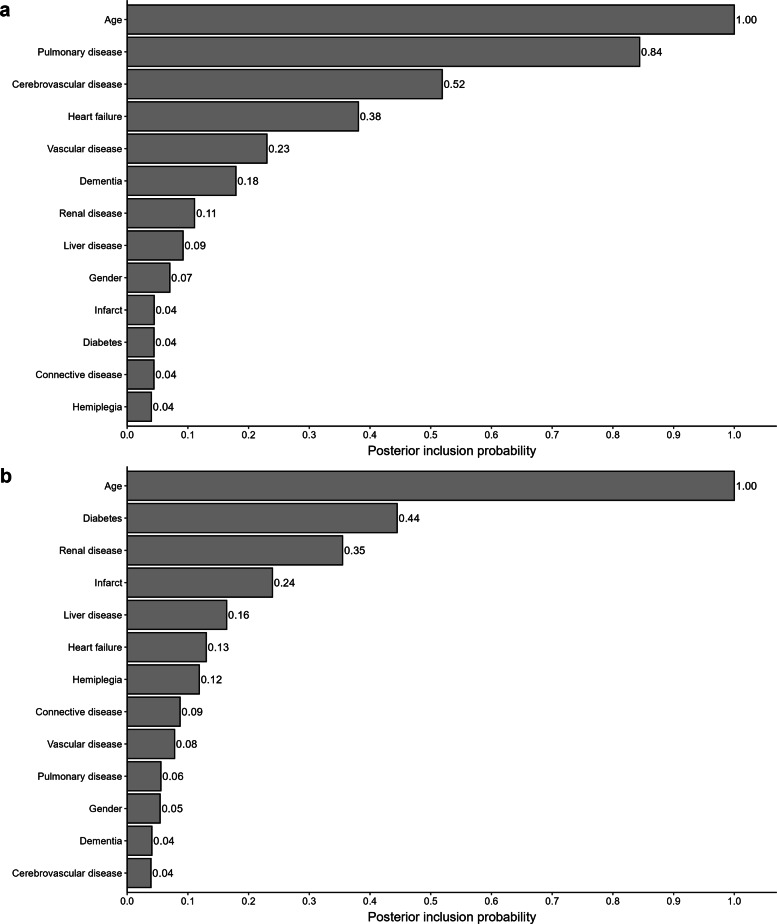
**a** Posterior Inclusion Probability for colorectal cancer patients with: **a** Stages I-III; **b** Stage IV

Figure 1a shows the results from the Bayesian variable selection step (Step 1) for age, sex, and ten comorbidities among CRC patients with stages I-III. The variables with a PIP higher than 0.5 were age, pulmonary disease, and cerebrovascular disease. However, among CRC patients with stage IV, the higher PIP was for age, diabetes, and renal disease (1.00, 0.44, and 0.35 PIP respectively, Fig. 1b).

Based on the results from the Bayesian variable importance, we fitted several Bayesian survival models based on both the general hazard (GH) structure (see Eq. 2) and proportional hazards (PH), which is a particular case of GH, as discussed in Step 2: modelling using a Bayesian parametric Hazard regression section. For each of these structures, different baseline hazard functions are specified, such as Log-Normal (LN), Log-Logistic (LL), and 3-parameter Power Generalised Weibull (PGW).

For CRC patients with stages I-III, the proposed models were compared using posterior model probabilities (see Supplementary Table 2). The best model was the PH model with LL baseline hazard including age, cerebrovascular disease, and COPD as covariates. Table 1 shows the posterior mean, hazard ratio (HR) and 95% credible intervals (CI), and the probability of an HR >1 given the data for the LL model. CRC patients with stages I-III and cerebrovascular disease had approximately two times higher (95% CI: 1.38 – 3.02) risk of death at 6 years after CRC diagnosis compared to CRC patients without that comorbidity (Table 1).

**Table 1:** Posterior summary for the proportional hazard model with the Log-Logistic (LL) baseline hazard specification for the CRC data considering stages I-III cancer patients in Spain, *n*=770

Interpretation	Parameter	Posterior Mean	HR	CI 2.5%	CI 97.5%	P(HR>1∣Data)
Age	*β*_1_	0.733	2.081	1.749	2.464	1.000
Cerebrovascular disease	*β*_2_	0.735	2.085	1.379	3.023	1.000
COPD	*β*_3_	0.623	1.865	1.401	2.446	1.000
LL scale	*η*	29.244	–	20.436	42.349	–
LL shape	*ν*	0.695	–	0.616	0.777	–

The best model for CRC patients with stage IV was the PH model with PGW baseline hazard including age and diabetes as covariates (see Supplementary Table 2). Table 2 shows the posterior mean, HR and 95% CI, and the probability of an HR >1 given the data for the PGW PH model. CRC patients with stage IV and diabetes had approximately 57% (95% CI: 1.15 – 2.11) higher risk of death at 6 years after CRC diagnosis compared to CRC patients without that diabetes (Table 2).

**Table 2:** Posterior summary for the proportional hazard model with the 3-parameter Power Generalised Weibull (PGW) baseline hazard specification for the CRC data considering stage IV cancer patients in Spain, *n*=287

Interpretation	Parameter	Posterior Mean	HR	CI 2.5%	CI 97.5%	P(HR>1∣Data)
Age	*β*_1_	0.522	1.685	1.439	1.973	1.000
Diabetes	*β*_2_	0.449	1.567	1.147	2.113	1.000
PGW scale	*η*	3.289	–	0.679	13.317	–
PGW shape 1	*ν*	0.793	–	0.658	0.964	–
PGW shape 2	*δ*	0.931	–	0.341	1.719	–

**Fig. 2:**
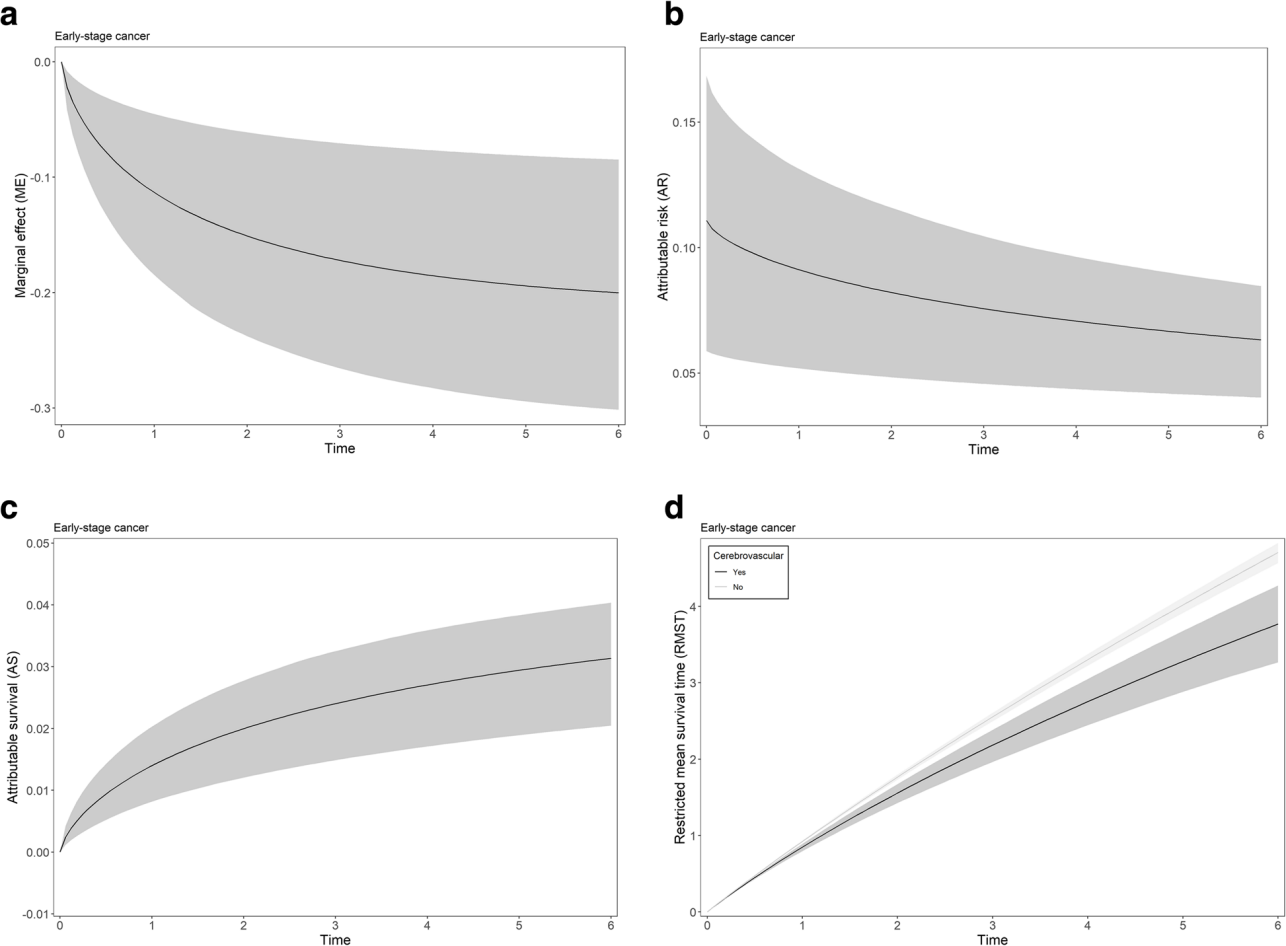
Posterior predictive **a** marginal effect (ME), **b** attributable risk (AR), **c** attributable survival (AS), **d** restricted mean survival time (RMST), and their respective 95% credible intervals for cerebrovascular disease in early-stage cancer patients from CRC data, using the proportional hazard model with the Log-Logistic (LL) baseline hazard specification

Figure 2 shows the PPME (a), PPAR (b), PPAS (c), and the RMST (d), including their respective 95% CI for cerebrovascular disease in CRC with stages I-III and derived from the LL PH model. Overall the PPME and PPAR decreased while the portion of detrimental outcome rate attributable (PPAR) to cerebrovascular disease increased over time (Fig. 2 a,b,c). The RMST for CRC patients with the cerebrovascular disease was consistently smaller than for patients without this comorbidity (Fig. 2d). Supplementary Figs. 1 and 2 show the summary measures for the other (dichotomous) variables from LL PH (early-stage = I-III) and PGW PH (late-stage = IV) models for CRC data.

### Results: lung data analysis by stage

There were 1,259 lung cancer patients. Among them, 16.6% were female, and more than half of the patients were diagnosed with stage IV (54.7%), but the proportion of lung cancer women with stage IV was markedly smaller than among men (i.e., 18.0% vs. 81.9%). The median age at lung cancer diagnosis was 69 years (IQR: 18 years). The pattern of comorbidities was similar by lung cancer stage. The most common comorbidities among stages I-III lung cancer patients were COPD (42.5%), diabetes (21.1%), and heart failure (17.7%), and COPD (31.5%), diabetes (20.4%), and heart failure (15.7%) among stage IV. There was 4.4% of missing stage and 12.9% for smoking status (Supplementary Table 3).

**Fig. 3:**
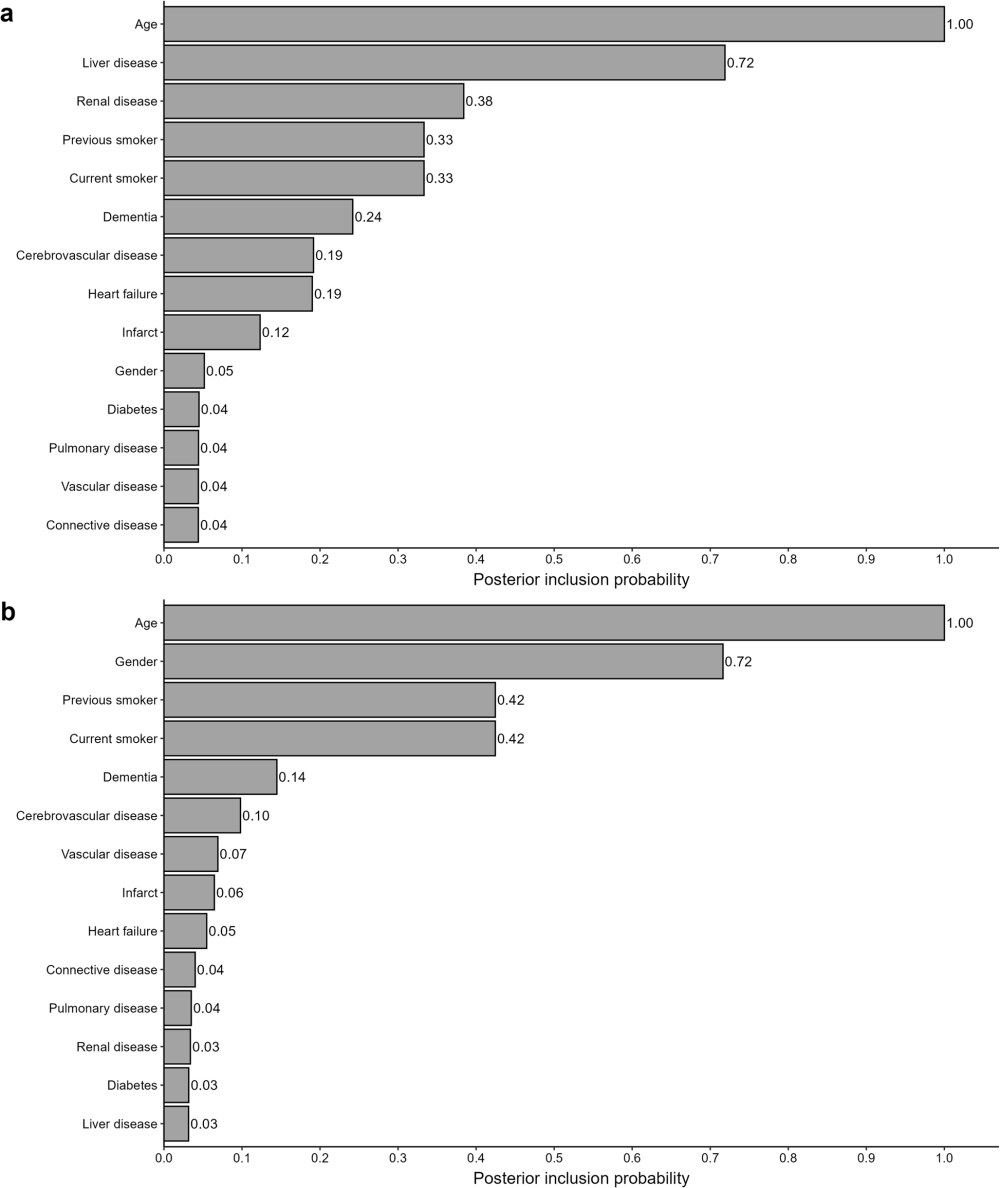
**a** Lung - Stages I-III: Posterior Inclusion Probability (PIP); **b** Lung - Stage IV: PIP

Figure 3a shows the results from the Bayesian variable importance selection for age, sex, and ten comorbidities among lung cancer patients with stages I-III. Only age and liver disease showed a PIP >0.5, but renal disease, smoking status, and dementia showed PIPs >0.2. However, among lung cancer patients with stage IV, the higher PIP was for age, gender, and previous smoking status (1.00, 0.72, and 0.42 PIP respectively) (Fig. 3b).

Based on the results from the Bayesian variable importance, we fitted several Bayesian survival models based on both the general hazard (GH) structure (see Eq. 2) and PH, which is a particular case of GH. For each of these structures, different baseline hazard functions are specified, such as Log-Normal (LN), Log-Logistic (LL), and 3-parameter Power Generalised Weibull (PGW).

For lung patients with stages I-III, the proposed models were compared using posterior model probabilities (see Supplementary Table 4). The best model was the PH model with LL baseline hazard including age, smoking status, dementia, renal and liver disease as covariates. Table 3 shows the posterior mean, hazard ratio (HR) and 95% credible intervals (CI), and the probability of an HR >1 given the data for the LL model. Lung cancer patients with stages I-III and current smokers had approximately two times higher (95% CI: 1.43 – 2.86) risk of death at 8 years after a cancer diagnosis than lung cancer patients without that comorbidity. Furthermore, compared to lung cancer patients without dementia, those affected for that comorbidity showed an 85% increased risk of death at 8 years after cancer diagnosis (HR: 1.85, 95% CI: 1.08 – 3.05) (Table 3).

**Table 3:** Posterior summary for the proportional hazard model with the Log-Logistic (LL) baseline hazard specification for the lung data considering stage I-III cancer patients in Spain, *n*=566

Interpretation	Parameter	Posterior Mean	HR	CI 2.5%	CI 97.5%	P(HR>1∣Data)
Age	*β*_1_	0.439	1.551	1.382	1.751	1.000
Previous smoker	*β*_2_	0.393	1.481	1.071	2.100	0.992
Current smoker	*β*_3_	0.699	2.012	1.426	2.863	1.000
Dementia	*β*_4_	0.613	1.846	1.077	3.048	0.989
Renal disease	*β*_5_	0.512	1.669	1.122	2.413	0.994
Liver disease	*β*_6_	0.518	1.679	1.088	2.509	0.990
LL scale	*η*	3.221	–	1.988	5.112	–
LL shape	*ν*	0.943	–	0.862	1.035	–

The best model for lung patients with stage IV was the PH model with PGW baseline hazard including age, sex, and smoking status as covariates (see Supplementary Table 4). Table 4 shows the posterior mean, HR and 95% CI, and the probability of an HR >1 given the data for the PGW proportional hazard model. Lung cancer patients with stage IV and current smokers had approximately 72% (95% CI: 1.28 – 2.31) higher risk of death at 6 years after cancer diagnosis compared to non-smoker lung cancer patients (Table 4).

**Table 4:** Posterior summary for the proportional hazard model with the 3-parameter Power Generalised Weibull (PGW) baseline hazard specification for the lung data considering stage IV cancer patients in Spain, *n*=693

Interpretation	Parameter	Posterior Mean	HR	CI 2.5%	CI 97.5%	P(HR>1∣Data)
Age	*β*_1_	0.309	1.362	1.243	1.501	1.000
Female vs. male	*β*_2_	0.170	1.185	0.928	1.543	0.910
Previous smoker	*β*_3_	0.428	1.534	1.132	2.054	0.999
Current smoker	*β*_4_	0.542	1.719	1.284	2.309	1.000
PGW scale	*η*	0.336	–	0.198	0.581	–
PGW shape 1	*ν*	1.150	–	0.993	1.324	–
PGW shape 2	*δ*	2.245	–	1.550	3.049	–

**Fig. 4:**
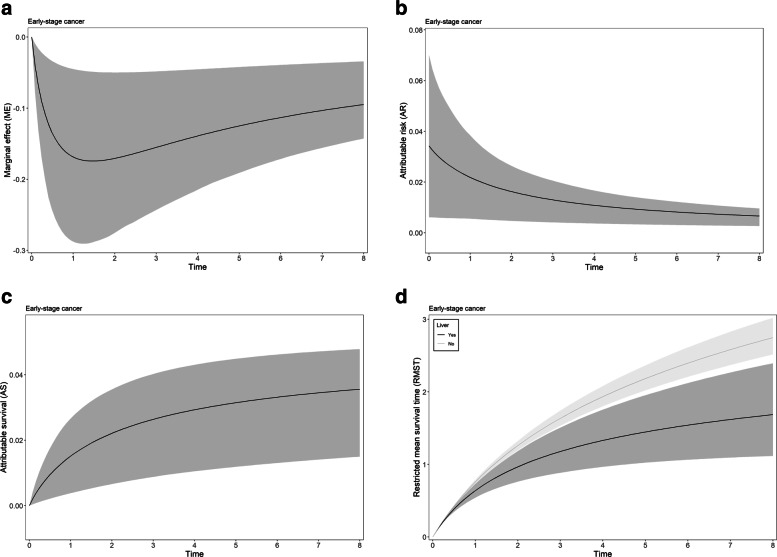
Posterior predictive **a** marginal effect (ME), **b** attributable risk (AR), **c** attributable survival (AS), **d** restricted mean survival time (RMST), and their respective 95% credible intervals for current liver disease in early-stage cancer patients from lung data, using the proportional hazard model with the Log-Logistic (LL) baseline hazard specification

Figure 4 shows the PPME (a), PPAR (b), PPAS (c), and the RMST (d), including their respective 95% CI for current liver disease status in lung cancer patients with stages I-III and derived from the LL proportional hazard model. Overall the PPME and PPAR decreased while the portion of detrimental outcome rate attributable (PPAR) to liver disease increased over time (Fig. 4a, b, c). The RMST for lung cancer patients with liver disease was consistently smaller than for patients without this comorbidity (Fig. 4d). The most critical comorbid conditions we identified were liver and renal diseases and smoking among lung cancer patients. Supplementary Figs. 3 and 4 show the summary measures for the other (dichotomous) variables from LL PH (early-stage = I-III) and PGW PH (late-stage = IV) models for lung data.

## Discussion

We have developed a principled three-step approach to select the comorbidities that affect cancer patients’ survival probability and quantity of their effect. We have adopted a Bayesian framework in all steps as this allows us to quantify uncertainty about the selected models and easily obtain interval inferences about quantities of interest. We have made some choices about the specific methods used for variable selection, survival modelling, and effect measures. However, one of the strengths of the proposed approach is that one could opt for alternative methods on each step. For instance, for Step 1, several variable selection methods could be used instead, and we refer the reader to Rubio and Rossell [8] for an extensive literature review on these methods. Regarding Step 2, Bayesian survival modelling is open to the use of other hazard structures and semiparametric methods. For Step 3, we point out that alternative model summaries and effect measures in survival analysis to those presented here can be included in the analysis. We argue that our work will provide cancer epidemiologists, applied researchers, and other stakeholders with the tools to conduct thorough analyses on the effect of comorbidities on cancer prognosis and survival and to foster other developments in this area.

A common approach to assess the effect of comorbidity on cancer survival includes weighted scores such as the Charlson comorbidity index [33]. We argue that the approach presented here can provide a more insightful clinical utility for identifying the most important comorbidities and their effect on cancer survival. For instance, in our applications, we show how to identify vulnerable groups of patients who have combinations of comorbidities that markedly affect the population’s survival probability. This information can inform policy-makers who can develop targeted strategies to improve cancer survival.

Since we have adopted a parametric modelling approach, the calculation of the different marginal and conditional summary measures automatically adjusts for other covariates. Nonetheless, a potential limitation of our applications, which is indeed endemic of population studies, is unmeasured confounders or covariates. For instance, there may be other comorbidities that were not measured or available for research, which may bias the estimates of such measures. A possible solution for this problem is the use of individual frailty models, which can account for individual unobserved heterogeneity. The study of such models and the effect of individual unobserved heterogeneity on these measures represents a future research direction the authors have already considered. Moreover, epidemiologists may also be interested in calculating standardised effect measures (such as age-standardized measures, where the effect measures are weighted averages of the age-specific groups). The proposed methods remain valid under the typical standardisation methods of interest in practice.

We have found that different combinations of comorbidities affect the survival of cancer patients with different types of cancers and tumour stages, a seemingly novel result with implications for clinicians and policymakers. For instance, we identify age, cerebrovascular, and COPD as risk factors for shorter survival among stages I-III CRC patients and age and diabetes for stage IV CRC patients. Our results are consistent with previous evidence showing that cerebrovascular and COPD comorbidities may delay or modify treatment alternatives among CRC patients. Thus it may explain the higher risk of death and shorter CRC survival [34]. Diabetes appears to increase the risk for primary cancer recurrence [1]. The association between diabetes and shorter CRC survival we found among patients with stage IV is consistent with previous evidence [35]. It has been shown that diabetes can hide or modify cancer symptoms, thus delaying cancer diagnosis [1]. Interestingly age, smoking, dementia, renal and liver disease were associated with shorter survival among stages I-III lung cancer patients, and age, sex, and smoking among stage IV lung cancer patients. Some of these comorbidities are related to preventive lifestyle behaviors such as smoking and drinking. Smoking contributes to over 80% of lung cancers in high-income countries [36]. This information has clinical value as well as it sheds light on the interplay of comorbidities with lung and colorectal cancer and their effect on population survival.

We acknowledge the limited generalisability of the illustration, as it only included data from two population-based cancer registries. A natural extension of our work consists of analyzing the most incident cancer sites at a national level for other countries. Information about comorbidities has only recently become available at the population level in several countries. Results must be interpreted cautiously as we merged stages I-III vs. IV in our illustrative examples. We aimed to produce results for metastasised vs. non-metastasised tumours, which implies a meaningful clinical contrast. Furthermore, there was a different distribution of comorbidities for both groups. However, this stratification is open to debate as it could be argued that stage III differs biologically and clinically from stage I and cannot be considered an early stage. Overall, data stratification should be based on clinical information to allow the end-user to produce interpretable results. Furthermore, we have compared the results obtained in Step 1 (Bayesian variable selection) with those obtained using Cox-LASSO, implemented in the R-package glmnet (version 4.1-2) [37]. The results are similar, albeit, Cox-LASSO is sensitive to the choice of the penalty parameter: using the value lambda.min leads to the inclusion of more variables than those selected with lambda.1se. This emphasises the adequacy of a methodology that quantifies uncertainty in variable selection. With a global aging population, comorbidities are expected to increase [38]. We emphasise that the selected comorbidities represent those that affect the population survival. Thus, the discarded comorbidities may still play an important role at the individual level, but they do not significantly reduce survival at the population level. Finally, we have performed a sensitivity analysis of the effect of missing data (stage), assuming that the data are missing randomly. The study of other types of missing data mechanisms is of interest, but this is beyond the aims of this project.

## Data Availability

The data that support the findings of this study are available from the Regional Government of Andalusia and the Andalusian Health Department. Still, restrictions apply to the availability of these data, which is often the case with cancer registry data, and so are not publicly available. The Regional Government of Andalusia and the Andalusian Health Department should be contacted to access the raw data from the present study. The code and packages used in the article are made available at a GitHub repository: https://github.com/migariane/BayesVarImpComorbiCancer.

## Abbreviations

AFT: Accelerated Failure Time AH: Accelerated Hazard AIC: Akaike Information Criteria BIC: Bayesian Information Criteria DIC: Deviance Information Criteria CI: Credible Interval COPD: Chronic obstructive pulmonary disease CRC: Colorectal Cancer CS: Posterior predictive conditional survival function GH: General Hazard LL: Log-logistic LN: Log-normal MS: Posterior predictive marginal survival function PPAR: Posterior predictive attributable risk function PPAS: Posterior predictive attributable survival function PPCE: Posterior predictive conditional effect PGW: Power Generalized Weibull PPME: Posterior predictive marginal effect PH: Proportional Hazard PIP: Posterior Inclusion Probability RMST: Restricted mean survival time
